# An Update to the Pilot Study of ^177^Lu-PSMA in Low Volume Hormone-Sensitive Prostate Cancer

**DOI:** 10.3389/fnume.2022.863101

**Published:** 2022-05-03

**Authors:** Bastiaan M. Privé, Constantijn H. J. Muselaers, Inge M. van Oort, Marcel J. R. Janssen, Steffie M. B. Peters, Willemijn A. M. van Gemert, Maike J. M. Uijen, Melline M. G. Schilham, J. P. Michiel Sedelaar, Harm Westdorp, Niven Mehra, Martin Gotthardt, Jelle O. Barentsz, Winald R. Gerritsen, J. Alfred Witjes, James Nagarajah

**Affiliations:** ^1^Department of Radiology and Nuclear Medicine, Radboudumc, Nijmegen, Netherlands; ^2^Department of Urology, Radboudumc, Nijmegen, Netherlands; ^3^Department of Medical Oncology, Radboudumc, Nijmegen, Netherlands

**Keywords:** hormone sensitive, prostate cancer, lutetium-177-PSMA-617, radioligand therapy, urologic oncology, metastases-directed therapies

## Abstract

^177^Lu-PSMA-617 radioligand therapy is a novel treatment for end-stage prostate cancer, which could also be applied to patients with hormone-sensitive prostate cancer with high expression levels of prostate-specific membrane antigen (PSMA). In this perspective, we review the recent results of toxicity, radiation doses, and treatment effect of ^177^Lu-PSMA in patients with low volume metastatic hormone-sensitive prostate cancer. Moreover, we present long-term follow-up data, such as toxicity and time without androgen deprivation therapy (ADT), of the patients who participated in this trial. Overall, ^177^Lu-PSMA appeared to be a feasible and safe treatment modality in this setting, as well as in long-term follow-up. We observed that men with a prostate-specific antigen (PSA) response of more than 50% seemed to especially benefit from this therapy by postponing ADT and thus preserving the quality of life.

## Perspective

Between 27 and 53% of patients with prostate cancer undergoing radical surgery or external beam radiotherapy (EBRT) will develop disease recurrence (1). If salvage surgery or EBRT is no option, androgen deprivation therapy (ADT) is recommended, particularly in patients with high prostate-specific antigen (PSA) velocity (e.g., PSA doubling time <6 months) (1). Despite favorable responses to ADT and novel drug combinations, there is an increasing interest in metastases-directed therapies (MDT) for oligometastatic disease, mainly because these treatments can postpone ADT-related side effects and thus preserve a good quality of life (1–4). Therefore, there is a need for more treatment options to control recurrent tumor progression while maintaining a good quality of life.

[^177^Lu]Lu-PSMA-617 (^177^Lu-PSMA) radioligand therapy is a novel treatment for patients with end-stage castrate-resistant prostate cancer (mCRPC) with promising efficacy and acceptable toxicity profile (5–8). This has resulted in an international registration trial for use of ^177^Lu-PSMA in patients with mCRPC, which recently reported positive outcomes with a 30–40% reduction in death from any cause (9). However, ^177^Lu-PSMA was yet unexplored in the (metastatic) hormone-sensitive setting (mHSPC). Recently, we evaluated if ^177^Lu-PSMA could become a potential effective MDT for patients with mHSPC harboring low tumor load (≤ 10 metastases on [^68^Ga]Ga-PSMA-PET imaging [PSMA-PET]) in a prospective pilot study (10). This article reports on long-term follow-up data, including toxicity, progression-free survival, and time without ADT.

All study procedures and in- and exclusion criteria were previously described (10). In short, men (age > 50 years) with histologically proven prostate cancer (PCa) and progressive disease after local therapy (PSA > 0.2 μg/l), with a PSA doubling time of <6 months and no curable treatment options left (e.g., surgery or external beam radiotherapy), were eligible for this trial. Moreover, patients needed to have low volume metastatic disease (≥1 but ≤ 10 positive lesions) on PSMA-PET with high tumor prostate-specific membrane antigen (PSMA) uptake. At the start of the study, none of the patients were allowed to use ADT. The study was approved by the Medical Review Ethics Committee Region Arnhem-Nijmegen (NL62774.091.17), was registered on clinicaltrials.gov (NCT03828838), and performed in accordance with the principles of Good Clinical Practice and the Declaration of Helsinki. The included patients received two cycles containing 3 and ~6 GBq ^177^Lu-PSMA 8 weeks in between. Patients were monitored up to 6 months after the last cycle. After the study, patients had regular appointments with their treating oncologist, including laboratory testing for PSA. The progression-free survival was reported following Prostate Cancer Working Group (PCWG3), wherein the time from the start of therapy to the date of first PSA had an increase of ≥25% and ≥2 ng/ml from nadir or date of start systemic treatment.

**Figure 1:**
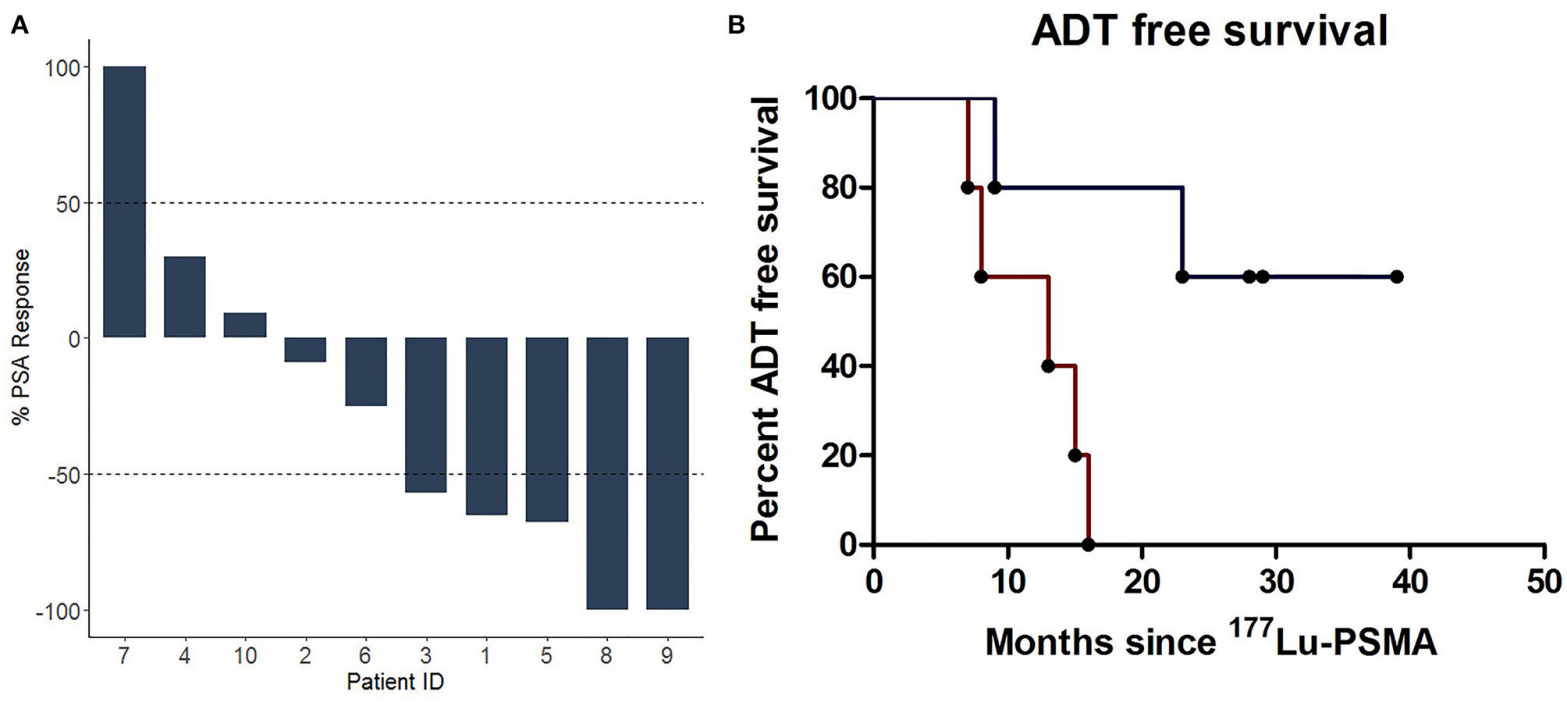
**(A)** Waterfall plot of the best prostate-specific antigen (PSA) response after two cycles of ^177^Lu-PSMA. **(B)** Kaplan-Meier plot showing the time without testosterone suppressing drugs (e.g., dutasteride, bicalutamide, LHRH agonists/antagists, enzalutamide, and abiraterone) grouped according to their PSA response. ADT, androgen deprivation therapy; LHRH, gonadotropin-releasing hormone; PSA, Prostate Specific Antigen; PSMA, Prostate Specific Membrane Antigen.

Since the start of treatment with ^177^Lu-PSMA-617, the median long-term follow-up of the cohort was 28 months (range 11–39 months). At this time, the median progression-free survival was 11 months (range 4–39 months). In line with this, the median androgen deprivation-free survival of the studied cohort was 16 months (range 7–39 months). Importantly, three of the ten patients are still postponing ADT (median 29 months [range 28–39 months]) (Figure 1). Prior to inclusion, all patients had PSA-doubling time <6 months. However, all of the patients showed a stabilization of the PSA-increase velocity following [^177^Lu]Lu-PSMA-617, with five of ten patients achieving a PSA decline of > 50% (Figure 1). The five patients with a >50% PSA response showed a longer ADT-free survival compared to patients with a <50% PSA response (Figure 1).

Following the two cycles of ^177^Lu-PSMA, none of the ten patients had severe treatment-related toxicities, and even the grades I-II toxicities (e.g., fatigue) recovered within a few weeks (10). Importantly, only mild and transient xerostomia was reported. Additionally, during long-term follow-up, none of the patients developed a dry mouth. However, one patient died 11 months after the study due to a cerebral vascular incident. This was deemed unrelated to ^177^Lu-PSMA-617 but may have been associated with the ADT that was started following the study. No clinically relevant changes in quality of life were observed applying a standardized questionnaire (EORTC QLQ-C30) before and after treatment of ^177^Lu-PSMA-617. In accordance with these outcomes, the dosimetry showed that patients could receive higher doses of ^177^Lu-PSMA and up to 38 GBq of ^177^Lu-PSMA-617 before organ-related toxicity occurred in this early setting (11). Importantly, doses to tumor lesions were consistently higher compared to the doses to the organs at risk (salivary glands, kidneys, and bone marrow).

Although the study consisted of a small cohort of selected patients, the results suggest a favorable outcome after ^177^Lu-PSMA in approximately half of the patients. These findings have encouraged us to initiate a larger prospective randomized multicenter study to provide stronger evidence for first-line ^177^Lu-PSMA in patients with oligometastatic mHSPC (e.g., NCT04443062) (12).

To conclude, ^177^Lu-PSMA appeared to be a feasible and safe treatment modality in low volume metastatic hormone-sensitive prostate cancer patients, also at long-term follow-up. In particular, those men with a PSA response of more than 50% seemed to benefit from this therapy by postponing ADT and preserving good quality of life.

## Data Availability Statement

The data that support the findings of this study are available from the corresponding author upon reasonable request.

## Ethics Statement

The studies involving human participants were reviewed and approved by Medical Review Ethics Committee Arnhem-Nijmegen. The patients/participants provided their written informed consent to participate in this study.

## Author Contributions

BP, CM, JW, and JN: conceptualization. BP, SP, CM, IO, MJ, WG, NM, JB, MG, JW, and JN: methodology. BP, SP, and JN: formal analysis and data curation. BP, CM, IO, MJ, MU, MS, WGem, HW, NM, JB, MG, JW, and JN: resources. BP and JN: writing—original draft preparation and visualization. BP, CM, IO, SP, MJ, JS, HW, NM, WGem, JB, MG, JW, and JN: writing—review and editing. WGer, JW, and JN: supervision. BP, SP, CM, and JN: project administration. MJ, WGer, JW, and JN: funding acquisition. All authors contributed to the article and approved the submitted version.

## Funding

This study was funded by the Radboud Oncology Foundation and the Dutch Prostate Cancer Foundation (Prostaatkankerstichting).

## Conflict of Interest

The authors declare that the research was conducted in the absence of any commercial or financial relationships that could be construed as a potential conflict of interest.

## Publisher's Note

All claims expressed in this article are solely those of the authors and do not necessarily represent those of their affiliated organizations, or those of the publisher, the editors and the reviewers. Any product that may be evaluated in this article, or claim that may be made by its manufacturer, is not guaranteed or endorsed by the publisher.

## References

[1] Cornford P van den Bergh RCN Briers E Van den Broeck T Cumberbatch MG De Santis M. EAU-EANM-ESTRO-ESUR-SIOG Guidelines on Prostate Cancer. Part II-2014-2020 Update: Treatment of Relapsing and Metastatic Prostate Cancer. Eur Urol. (2021) 79:263–82. 10.1016/j.eururo.2020.09.046 33039206

[2] Ost P Reynders D Decaestecker K Fonteyne V Lumen N De Bruycker A. Surveillance or metastasis-directed therapy for oligometastatic prostate cancer recurrence: a prospective, randomized, multicenter phase II trial. J Clin Oncol. (2018) 36:446–53. 10.1200/JCO.2017.75.4853 29240541

[3] Isbarn H Boccon-Gibod L Carroll PR Montorsi F Schulman C Smith MR. Androgen deprivation therapy for the treatment of prostate cancer: consider both benefits and risks. Eur Urol. (2009) 55:62–75. 10.1016/j.eururo.2008.10.008 18945543 PMC3090670

[4] Phillips R Shi WY Deek M Radwan N Lim SJ Antonarakis ES. Outcomes of observation vs stereotactic ablative radiation for oligometastatic prostate cancer: the ORIOLE phase 2 randomized clinical trial. JAMA Oncol. (2020) 6:650–9. 10.1001/jamaoncol.2020.0147 32215577 PMC7225913

[5] Hofman MS Violet J Hicks RJ Ferdinandus J Thang SP Akhurst T. [(177)Lu]-PSMA-617 radionuclide treatment in patients with metastatic castration-resistant prostate cancer (LuPSMA trial): a single-centre, single-arm, phase 2 study. Lancet Oncol. (2018) 19:825–33. 10.1016/S1470-2045(18)30198-0 29752180

[6] Heck MM Tauber R Schwaiger S Retz M D'Alessandria C Maurer T. Treatment outcome, toxicity, and predictive factors for radioligand therapy with ^177^Lu-PSMA-I T in metastatic castration-resistant prostate cancer. Eur Urol. (2019) 75:920–6. 10.1016/j.eururo.2018.11.016 30473431

[7] Hofman MS Emmett L Sandhu S Iravani A Joshua AM Goh JC. [^177^Lu]Lu-PSMA-617 versus cabazitaxel in patients with metastatic castration-resistant prostate cancer (TheraP): a randomised, open-label, phase 2 trial. Lancet. (2021) 397:797–804. 10.1016/S0140-6736(21)00237-3 33581798

[8] Privé BM Slootbeek PHJ Laarhuis BI Naga SP van der Doelen MJ van Kalmthout LWM. Impact of DNA damage repair defects on response to PSMA radioligand therapy in metastatic castration-resistant prostate cancer. Prostate Cancer Prostatic Dis. (2021) 1–8. 10.1038/s41391-021-00424-2 34253846

[9] Sartor O de Bono J Chi KN Fizazi K Herrmann K Rahbar K. Lutetium-177–PSMA-617 for Metastatic Castration-Resistant Prostate Cancer. N Engl J Med. (2021). 10.1056/NEJMoa2107322 PMC8446332 34161051

[10] Privé BM Peters SMB Muselaers CHJ van Oort IM Janssen MJR Sedelaar M. Lutetium-177-PSMA-617 in low-volume hormone sensitive metastatic prostate cancer, a prospective pilot study. Clin Cancer Res. (2021) 27:3595–601. 10.1055/s-0040-1708448 33883176

[11] Peters SMB Privé BM de Bakker M de Lange F Jentzen W Eek A. Intra-therapeutic dosimetry of [177Lu]Lu-PSMA-617 in low-volume hormone-sensitive metastatic prostate cancer patients and correlation with treatment outcome. Eur J Nuclear Med Mol Imaging. (2020) 20:884. 10.1007/s00259-021-05471-4 34218300 PMC8803803

[12] Privé BM Janssen MJR van Oort IM Muselaers CHJ Jonker MA de Groot M. Lutetium-177-PSMA-I&T as metastases directed therapy in oligometastatic hormone sensitive prostate cancer, a randomized controlled trial. BMC Cancer. (2020) 20:884. 10.1186/s12885-020-07386-z 32928177 PMC7490874

